# Functional analysis reveals that *RBM10* mutations contribute to lung adenocarcinoma pathogenesis by deregulating splicing

**DOI:** 10.1038/srep40488

**Published:** 2017-01-16

**Authors:** Jiawei Zhao, Yue Sun, Yin Huang, Fan Song, Zengshu Huang, Yufang Bao, Ji Zuo, David Saffen, Zhen Shao, Wen Liu, Yongbo Wang

**Affiliations:** 1 Department of Cellular and Genetic Medicine, School of Basic Medical Sciences, Fudan University, Shanghai, 200032, China; 2 School of Life Sciences, Fudan University, Shanghai, 200438, China; 3 Institutes of Brain Science, Fudan University, Shanghai, 200032, China; 4 Key Laboratory of Computational Biology, CAS-MPG Partner Institute for Computational Biology, Shanghai Institutes for Biological Sciences, Chinese Academy of Sciences, Shanghai, 200031, China; 5 Shanghai Medical College, Fudan University, Shanghai, 200032, China; 6 State Key Laboratory for Medical Neurobiology, Fudan University, Shanghai, 200032, China

## Abstract

*RBM10* is an RNA splicing regulator that is frequently mutated in lung adenocarcinoma (LUAD) and has recently been proposed to be a cancer gene. How *RBM10* mutations observed in LUAD affect its normal functions, however, remains largely unknown. Here integrative analysis of *RBM10* mutation and RNA expression data revealed that LUAD-associated *RBM10* mutations exhibit a mutational spectrum similar to that of tumor suppressor genes. In addition, this analysis showed that *RBM10* mutations identified in LUAD patients lacking canonical oncogenes are associated with significantly reduced *RBM10* expression. To systematically investigate *RBM10* mutations, we developed an experimental pipeline for elucidating their functional effects. Among six representative LUAD-associated *RBM10* mutations, one nonsense and one frameshift mutation caused loss-of-function as expected, whereas four missense mutations differentially affected *RBM10*-mediated splicing. Importantly, changes in proliferation rates of LUAD-derived cells caused by these *RBM10* missense mutants correlated with alterations in RNA splicing of *RBM10* target genes. Together, our data implies that *RBM10* mutations contribute to LUAD pathogenesis, at least in large part, by deregulating splicing. The methods described in this study should be useful for analyzing mutations in additional cancer-associated RNA splicing regulators.

Large-scale sequencing and computational analysis have enabled systematic identification of genetic mutations in various types of cancer^1^,^2^,^3^,^4^,^5^. This information, in particular concerning certain oncogenes, has been successfully used for molecular classification of cancer subtypes, drug development and targeted therapies^6^. For example, identification of mutations in specific oncogenes has dramatically improved diagnosis and treatment for patients with breast cancer^7^ and non-small cell lung cancer (NSCLC)^8^,^9^. However, the functional and clinical significance of many or even most of the identified mutations in cancer remains unknown^1^. This lack of knowledge has impeded the development of new diagnostic and therapeutic targets for a large number of cancer patients.

Mutations of uncertain significance (MUS) include those in recently identified candidate cancer genes^10^,^11^ and rare mutations in *bona fide* oncogenes and tumor suppressor genes^12^,^13^,^14^,^15^. Although algorithms have been developed to predict the functional effects of mutations in cancer, they often produce inconsistent results^15^,^16^,^17^. It is particularly challenging to predict the consequences of missense mutations, and experiments are often required to establish their effects. Robust methods for functional characterization of MUS in cancer-associated genes are therefore needed to develop new clinical applications.

Lung adenocarcinoma (LUAD) is the most common subtype of lung cancer, a heterogeneous set of diseases responsible for the largest number of cancer-related death worldwide^8^. Although dramatic progress has been made in the application of targeted therapies for patients carrying specific oncogenic mutations, including *EGFR, BRAF, ERBB2* mutations and ALK, RET, ROS1 translocations, the majority of LUAD patients do not harbor these currently actionable mutations^8^,^10^. Recent sequencing efforts aimed at comprehensively identifying genetic alterations in LUAD have detected frequently occurring mutations in putative cancer genes, including proteins involved in RNA splicing and chromatin modification^10^,^18^. Elucidating the functional effects of these genetic mutations should facilitate clinical management of LUAD patients.

*RBM10* is an RNA binding protein and splicing regulator located on the X chromosome. Loss-of-function (LOF) mutations in *RBM10* have been reported to cause TARP (Talipes equinovarus, atrial septal defect, Robin sequence and persistent left superior vena cava, MIM #311900) syndrome, a congenital, multifaceted developmental disorder^19^,^20^,^21^. In addition, *RBM10* mutations have been frequently observed in LUAD, and less frequently in other types of cancers, including colorectal carcinoma^22^, pancreatic ductal adenocarcinoma (PDA)^23^ and intraductal papillary mucinous neoplasm (IPMN)^24^. Notably, *RBM10* mutations were found to be significantly enriched in LUAD in men compared with women^10^,^25^, suggesting that there may be gender-specific contributions to the development and progression of LUAD.

Our previous study revealed that *RBM10* promotes exon skipping in its target genes, including the cancer genes *NUMB* and *CREBBP*^26^. Other studies have demonstrated that *RBM10* suppresses LUAD cell proliferation by inhibiting the formation of the long splice variant of *NUMB*, which in turn represses NOTCH signaling activity^27^,^28^. It has also been shown that *RBM10* can promote apoptosis by modulating the gene expression of tumor necrosis factor alpha (TNF-α) in breast cancer and leukemia cell lines^29^. Accordingly, *RBM10* has recently been classified as a cancer gene and listed in the Catalog of Somatic Mutations in Cancer (COSMIC) database^30^. However, it remains largely unclear how *RBM10* mutations, especially missense mutations, affect *RBM10*-mediated regulation of alternative splicing and suppression of LUAD cell proliferation.

In this study, we systematically analyzed the mutational landscape and expression of *RBM10* in LUAD. We also designed a robust functional assessment pipeline and used this to elucidate the effects of six representative *RBM10* mutations in LUAD. Our findings revealed that *RBM10* mutations likely affect its tumor suppressive activity, at least in large part, by interfering with its splicing regulatory functions.

## Results

### *RBM10* mutational spectrum and expression in lung adenocarcinoma (LUAD)

**Figure 1:**
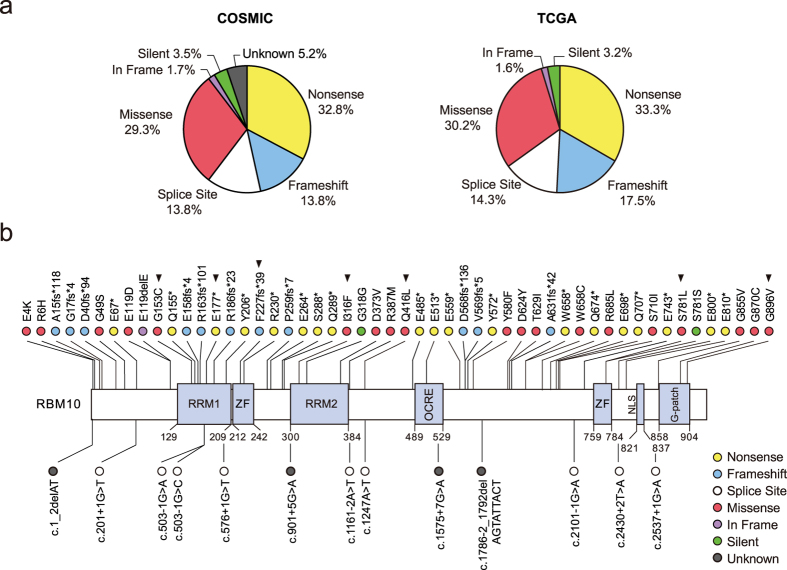
Mutational spectrum of *RBM10* in lung adenocarcinoma (LUAD). (**a**) Distributions of distinct types of *RBM10* mutations in LUAD samples in the total COSMIC collection and the TCGA cohort subset. Different mutation types are indicated by different colors. (**b**) Locations of COSMIC and TCGA LUAD *RBM10* mutations within the *RBM10* protein sequence. Characterized protein domains are represented by light purple boxes. Arrowheads indicate mutations chosen for experimental assays. RRM: RNA recognition motif; ZF: zinc finger; OCRE, Octamer Repeat; NLS: nuclear localization signals; G-patch: Glycine-patch. Data was downloaded from COSMIC (http://cancer.sanger.ac.uk/cosmic) and TCGA (http://cancergenome.nih.gov/).

*RBM10* mutations were identified in 53 of 1008 LUAD samples (~5.3%) listed in the COSMIC database, which compiles systematic screening data from The Cancer Genome Atlas (TCGA) and International Cancer Genome Consortium (ICGC) and manual curation data from independent studies. The mutational frequency was higher in TCGA LUAD cohort^10^,^18^, with *RBM10* mutations identified in 43 out of 546 patients (~7.9%). The majority of *RBM10* mutations were protein-truncating variants (PTVs), including nonsense, frameshift and splice site mutations, which often cause loss-of-function (LOF) (Fig. 1a). In addition to PTVs, a large fraction of *RBM10* mutations were missense mutations (~30%), for which functional consequences are often uncertain and require experimental confirmation (Fig. 1a). Our survey also showed that mutations occurred throughout the *RBM10* coding region rather than concentrating at specific sites (Fig. 1b). This mutational landscape resembles that observed in canonical tumor suppressive genes^2^.

**Figure 2:**
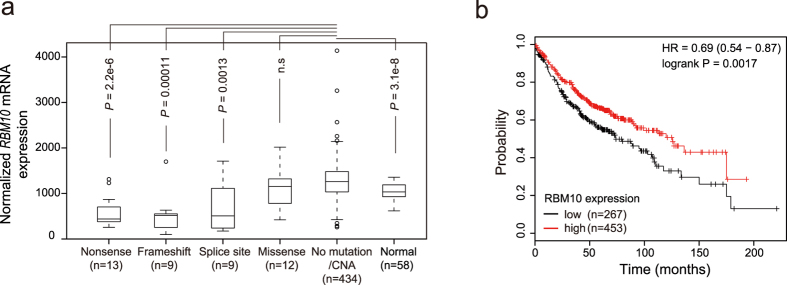
Effects of mutations on *RBM10* mRNA expression and the association of *RBM10* expression with LUAD patient survival. (**a**) *RBM10* mRNA expression in LUAD samples with different types of mutations, without mutation or copy number alteration (no mutation/CNA) and in tumor-adjacent normal tissues (Normal). Boxes represent the median (inside line) and the quartiles (upper and bottom boarder lines). “Whiskers” above and below the boxes represent maximum and minimum values within the 1.5 × IQ (inter-quarter) range. *P* values were calculated by Mann-Whitney tests followed by Benjamini & Hochberg corrections. Data from TCGA, see Methods for details. (**b**) Kaplan-Meier analysis of overall survival of LUAD patients, using data from the KM plotter database (http://www.kmplot.com). Patients were stratified into “low” and “high” *RBM10* RNA expression groups, based on the auto select best cutoff. *P* = 0.0017 (log rank test). HR: hazard ratio.

To examine how these mutations affect gene expression, we compared *RBM10* mRNA levels in LUAD samples harboring different types of *RBM10* mutations with LUAD samples lacking *RBM10* mutations and samples from tumor-adjacent normal tissues. For these comparisons, we focused on the TCGA samples, for which RNA sequencing (RNA-seq) data are publically accessible. To rule out possible confounding effects of copy number alterations (CNAs) (Supplementary Fig. S1), we removed samples with *RBM10* CNAs from our analysis. As shown in Fig. 2a, LUAD samples carrying three types of PTVs exhibited statistically significant reductions in mRNA expression compared to samples lacking *RBM10* mutations or CNAs (*P* = 2.2e-06, 0.00011, 0.0013 for nonsense, frameshift, and splice site mutations, respectively), or tumor-adjacent normal tissues (*P* = 0.00035, 0.00035, 0.029 for nonsense, frameshift, and splice site mutations, respectively). By contrast, samples harboring missense mutations did not show statistically significant reductions in *RBM10* mRNA levels. These results suggest that PTVs often cause *RBM10* LOF, but possible functional consequences of missense mutations cannot be deduced from RNA expression levels alone.

We next examined correlations between *RBM10* expression and overall survival of LUAD patients using an online Kaplan-Meier plotter^31^ (http://www.kmplot.com). This analysis revealed that high levels of *RBM10* mRNA expression correlated with longer overall survival (Fig. 2b), suggesting a possible suppressive role of *RBM10* in LUAD progression.

### Oncogene-negative *RBM10* mutations in LUAD patients correlate with significantly reduced *RBM10* expression

**Figure 3:**
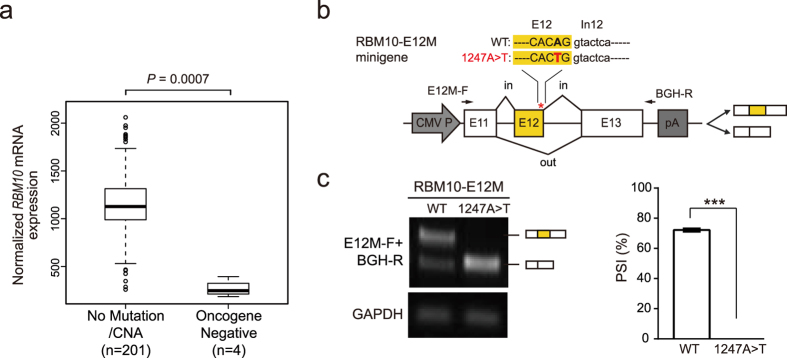
Oncogene-negative *RBM10* mutations, including one coding mutation located close to a splice site, associate with reduced *RBM10* mRNA expression. (**a**) *RBM10* mRNA expression in four samples harboring oncogene-negative *RBM10* mutations and 201 LUAD samples lacking *RBM10* mutations or CNAs (no mutation/CNA). Data obtained from a recently published LUAD cohort study by TCGA^10^. *P* = 0.0007 (Mann-Whitney test). (**b**) Structures of minigene splicing reporters for *RBM10* exon 12 wild type and mutant (*RBM10*-E12M-WT and -1247A>T, respectively). Arrows indicate target specific forward primer (E12M-F) and vector specific reverse primer (BGH-R) used in RT-PCR. Asterisk indicates the position of mutation. E: Exon, In: intron. (**c**) RT-PCR analysis of exon 12 inclusion levels for *RBM10*-E12M-WT and -1247A>T in HEK293 cells. Left panel: a representative gel image. *GAPDH*: internal control. The full-length gel image is presented in Supplementary Fig. S9a. Right panel: quantification of exon 12 inclusion levels using PSI (percent-spliced-in) values. Data present means ± SEM. n = 4 biological replicates. ***P ≤ 0.001 (Student’s *t*-test).

Mutations in putative cancer genes that occur in the absence of well-defined oncogenes are termed “oncogene-negative” mutations and are considered to be potential “drivers” of carcinogenesis^10^. To determine whether *RBM10* mutations occur in oncogene-negative patient samples, we examined mutation data from a recently published TCGA LUAD cohort study^10^. This analysis revealed that 4 of the 14 *RBM10* mutations reported in the 230 LUAD samples were oncogene-negative^10^. Interestingly, *RBM10* expression levels in each of the 4 oncogene-negative samples were significantly lower than that in LUAD samples lacking *RBM10* mutations or CNAs (Fig. 3a, *P* = 0.0007), suggesting these mutations cause LOF and contribute to LUAD pathogenesis. Inspection revealed that 3 of the 4 *RBM10* mutations found in oncogene-negative LUAD patients were unambiguously identified as PTVs, while the remaining one was annotated as a missense mutation (Q416L (c.1247A>T))^10^ (Supplementary Table S1).

### The oncogene-negative *RBM10* missense mutation disrupts *RBM10* splicing

To determine how the c.1247A>T mutation reduces expression of *RBM10*, we examined its DNA sequence context and found that the mutation is located in the second-to-last nucleotide of *RBM10* exon 12 (NM_005676.4). The A to T transversion reduced the 5′ splice site MAXENT score^32^ from 7.04 to 2.63, predicting decreased exon inclusion. To test this prediction, we generated a minigene splicing reporter, *RBM10*-E12M-WT, containing the entire genomic sequence from upstream exon 11 to downstream exon 13, and used site-directed mutagenesis to introduce the point mutation, producing *RBM10*-E12M-1247A>T (Fig. 3b). We independently transfected HEK293 cells with each of the two minigene reporters and examined exon 12 splicing levels using reverse transcription (RT)-PCR. Strikingly, WT construct showed approximately 71% inclusion of exon 12, estimated from PSI (percent-spliced-in) values of RT-PCR products, the point mutation completely abolished inclusion of exon 12 derived from the mutant construct (Fig. 3c).

*RBM10* exon 12 skipping produces a premature termination codon (PTC) positioned more than 55 nt upstream of the last exon-exon junction, and thus is predicted to trigger nonsense-mediated mRNA decay (NMD)^33^ and reduce *RBM10* mRNA expression level. These findings demonstrate that the main effect of the c.1247A>T missense mutation is not to produce a protein with an altered amino acid sequence, but rather significantly reduce *RBM10* expression. In fact, *RBM10* Q416L (c.1247A>T) overexpressed in HEK293 cells from a plasmid containing its coding sequence localized to the nucleus (Supplementary Fig. S5b) and produced splicing changes in four *RBM10* target genes similar to those observed following overexpression of *RBM10* WT (Supplementary Fig. S4). These observations suggest that the *RBM10* Q416L mutant protein itself has no effect on the splicing of *RBM10*-target genes. As this example demonstrates, caution is required when annotating coding mutations located close to splice sites.

### Pipeline for functional assessment of *RBM10* mutations

**Figure 4:**
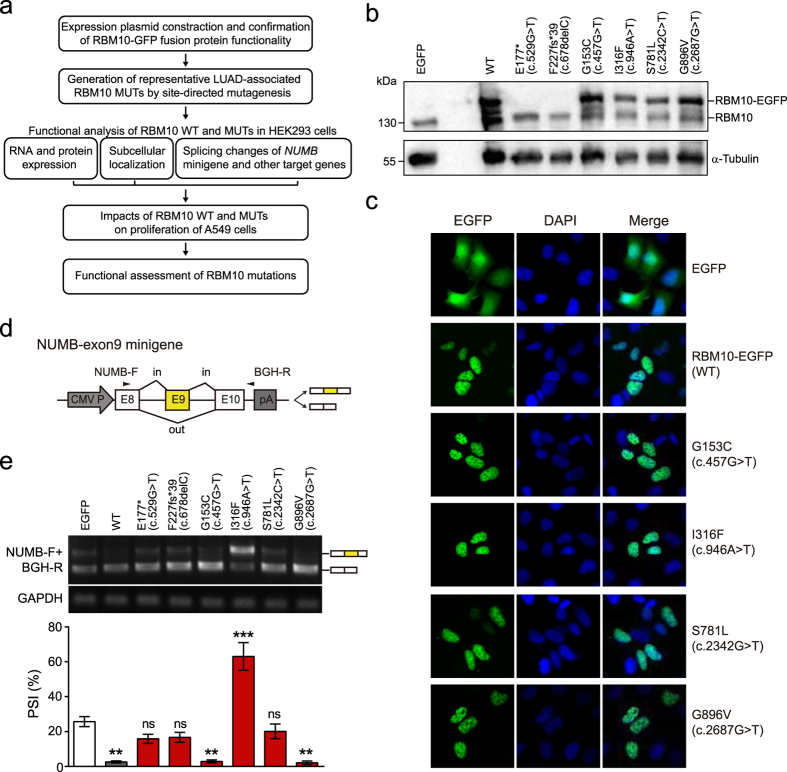
Protein expression and subcellular localization of *RBM10*-EGFP wild type (WT) and mutants (MUTs) and their effects on *NUMB* splicing in HEK293 cells. (**a**) Functional assessment pipeline. (**b**) Detection of endogenous *RBM10* and exogenous *RBM10*-EGFP WT and MUTs by western blot analysis using an antibody against *RBM10*. α-Tubulin: loading control. The full-length blot image is presented in Supplementary Fig. S9b. Results shown are a representative of three biological replicates. (**c**) Fluorescent images showing subcellular localizations of *RBM10*-EGFP WT and MUTs. Magnification: 40x. Scale bar: 10 μm. Nuclei were visualized by DAPI staining. (**d**) Structure of *NUMB* exon 9 minigene splicing reporter. Arrowheads represent target specific forward primer (NUMB-F) and vector specific reverse primer (BGH-R) used in RT-PCR. (**e**) Effects of *RBM10*-EGFP WT and MUTs on splicing of *NUMB* exon 9. Splice variants were detected by RT-PCR and the inclusion levels of exon 9 were quantified as PSI (percent-spliced-in) values. PSI means ± SEM from 3 independent experiments are plotted below the gel image. *GAPDH*: internal control. The full-length gel image is presented in Supplementary Fig. S9c. All conditions were compared to EGFP control. ns: not significant, **P ≤ 0.01, ***P ≤ 0.001 (One-way ANOVA followed by Dunnett’s test for multiple comparisons to a single control).

To systematically assess the effects of *RBM10* mutations, in particular missense mutations, we designed a functional assessment pipeline (Fig. 4a). Briefly, we constructed expression plasmids containing wild type (WT) or mutant (MUT) *RBM10* proteins with enhanced green fluorescent protein (EGFP) reporters fused to their C-termini. These plasmids were transiently transfected into HEK293 cells to assess the effects of the mutations on *RBM10* protein expression, subcellular localization and splicing regulatory functions. In addition, the effects of *RBM10* mutations on LUAD cell proliferation and RNA splicing of *RBM10* target genes were assessed in LUAD-derived A549 cells.

We applied the pipeline to the analysis of six *RBM10* mutations located in different conserved protein domains of *RBM10* (Fig. 1b) and predicted to be pathogenic by the FATHMM algorithm used in the COSMIC database^34^,^35^. The six mutations comprised: a nonsense mutation (E177* (c.529G>T)), a frameshift mutation (F227fs*39 (c.678delC)) and four missense mutations (G153C (c.457G>T), I316F (c.946A>T), S781L (c.2342C>T), G896V (c.2687G>T)).

Following transfection of HEK293 cells with *RBM10*-EGFP WT, the fusion protein was readily detected by western blot analysis (Fig. 4b) and predominantly localized to the nucleus (Fig. 4c). Overexpression (OE) of *RBM10*-EGFP WT in HEK293 cells also increased exon skipping in five known *RBM10* target genes (Supplementary Fig. S2), in agreement with previous studies^26^,^36^,^37^. These observations confirm that fusing EGFP to the C-terminus of *RBM10* does not interfere with its function, and thus demonstrate that *RBM10* fusion proteins can be used in functional assays.

### *RBM10* missense mutations have distinct effects on alternative splicing

Following independent transfection of *RBM10*-EGFP WT or MUT expression plasmids into HEK293 cells, no fusion protein or fluorescence was detected for the nonsense and frameshift MUTs (Fig. 4b and Supplementary Fig. S5a), consistent with the predicted disruption of protein expression. By contrast, each of the four *RBM10* missense fusion proteins had RNA and protein levels comparable to those of the WT fusion protein (Supplementary Fig. S3 and Fig. 4b) and localized primarily to the nucleus like the *RBM10* WT protein (Fig. 4c).

Because *RBM10* suppresses LUAD cell proliferation, at least partially by promoting exon 9 skipping of *NUMB*^27^,^28^, we performed *NUMB* minigene splicing reporter assays to explore the effects of the mutations on splicing of *RBM10* target genes (Fig. 4d). Compared with the EGFP control, cells transfected with *RBM10*-EGFP nonsense or frameshift MUT expression plasmids did not show significant changes in splicing of the *NUMB* minigene (Fig. 4e), consistent with the observed lack of expression of these fusion proteins (Fig. 4b). Intriguingly, OE of each of the four missense MUTs displayed distinct outcomes for *NUMB* exon 9 splicing. Similar to *RBM10* WT, OE of the G153C and G896V mutants produced significantly lower levels of exon 9 inclusion compared to the EGFP control (Fig. 4e, *P* ≤ 0.01), indicating that these mutations do not affect *RBM10*-regulated splicing. By contrast, OE of *RBM10*-S781L produced no significant difference compared to the EGFP control (Fig. 4e), indicating a LOF with regard to splicing. Of note, OE of *RBM10*-I316F produced significant higher levels of exon inclusion compared to the EGFP control, suggesting a change-of-function (Fig. 4e, *P* ≤ 0.001).

To further characterize the effects of OE of *RBM10* MUTs on splicing, we examined splicing changes of four *RBM10* target genes. OE of the above MUTs yielded results consistent with the trend obtained from *NUMB* minigene assays, except for *RBM10*-I316F, which produced no observable changes in splicing of the target genes compare to the EGFP control (Supplementary Fig. S4). This discrepancy may result from the low efficiency and heterogeneity of transient transfection assays, which produce mixtures of transfected and non-transfected cells. Together, these observations demonstrate that the two truncation *RBM10* mutations produced LOF, while the four missense mutations had differential effects on splicing, despite showing protein expression and subcellular localization patterns resembling *RBM10* WT.

### *RBM10* missense mutations exhibit correlated effects on LUAD cell proliferation and RNA splicing

To further investigate how the *RBM10* missense mutations affect LUAD cell proliferation, we used LUAD-derived A549 cells to construct stable tet-on cell lines inducibly expressing *RBM10*-EGFP WT or one of the three missense MUTs that showed distinct effects on splicing. Upon overexpression in A549 cells, each of the three mutant fusion proteins displayed nuclear localizations similar to *RBM10*-EGFP WT (Supplementary Fig. S6), consistent with observations in HEK293 cells (Fig. 4c).

**Figure 5:**
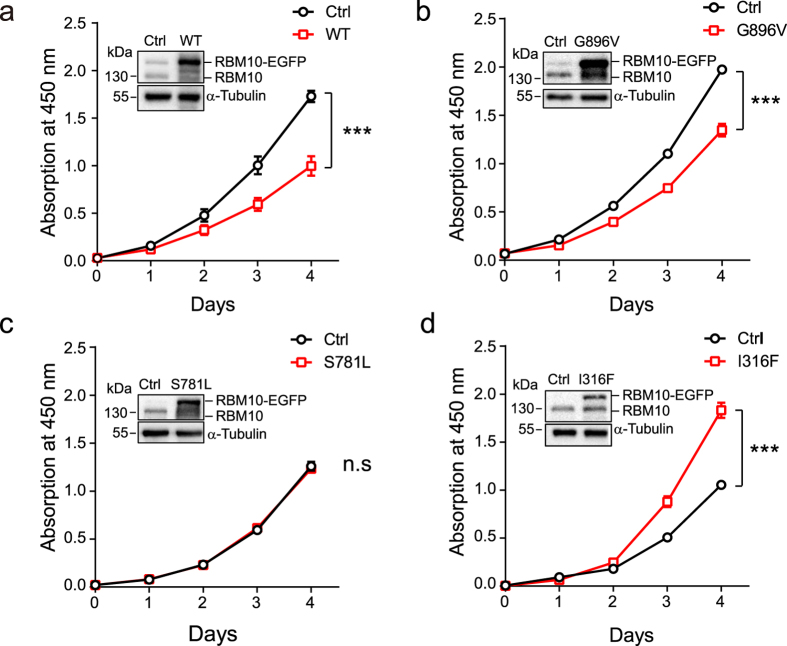
Effects of *RBM10* missense mutations on LUAD cell proliferation. (**a**–**d**) Growth curves of tet-on A549 cells inducibly overexpressing *RBM10*-EGFP wild type (**a**) or three missense mutants (**b**–**d**) using CCK-8 assays. Error bars indicate ± SEM, n = 3 biological replicates. ***P ≤ 0.001 (two-way ANOVA followed by Bonferroni correction for multiple testing). Insertions in the graphs show levels of endogenous *RBM10* and *RBM10*-EGFP proteins measured by western blot analysis using an antibody against *RBM10*. α-Tubulin: loading control. The full-length blot image is presented in Supplementary Fig. S9d. Fusion protein overexpression was achieved by 1 μg/ml doxycycline treatment (see Methods).

Cell counting kit-8 (CCK-8) assays revealed that OE of *RBM10*-EGFP WT significantly decreased the rate of cell proliferation (Fig. 5a, *P* ≤ 0.001), while *RBM10* depletion had the opposite effect (Supplementary Fig. S7). These results are consistent with previous findings that *RBM10* negatively regulates LUAD cell proliferation^27^,^28^. Among the three missense MUTs, expression of *RBM10*-EGFP G896V significantly decreased the rate of LUAD cell proliferation resembling WT (Fig. 5b, *P* ≤ 0.001), suggesting no alteration of function. Expression of *RBM10*-EGFP S781L caused no significant changes (Fig. 5c), indicating LOF. By contrast, expression of *RBM10*-EGFP 316 F significantly increased the rate of LUAD cell proliferation (Fig. 5d, *P* ≤ 0.001), indicating change-of-function.

Notably, the effects of these *RBM10* missense mutations on LUAD cell proliferation correlated with that on the splicing of *NUMB* exon 9 minigene reporter (Fig. 4e). Additionally, we investigated the effects of overexpressing *RBM10*-EGFP WT or MUTs on the splicing of four *RBM10* target genes in the tet-on A549 cells. We found that OE of *RBM10*-EGFP G896V enhanced skipping of each of four *RBM10*-regulated exons similar to *RBM10*-EGFP WT, OE of *RBM10*-EGFP S781L did not induce prominent changes in splicing of these exons, and OE of *RBM10*-EGFP I316F dramatically increased levels of exon inclusion in two of the four target genes: *NUMB* and *SAT1* (Supplementary Fig. S8). These results are in agreement with the trend obtained using the *NUMB* exon 9 minigene splicing reporter (Fig. 4e). The other two target genes, *CREBBP* and *POLDIP3*, did not show dramatic increases in exon inclusion levels upon *RBM10*-EGFP I316F OE, very likely because their basal inclusion levels were close to 100%, as observed in controls (Supplementary Fig. S8). Collectively, these observations indicate that the suppressive functions of *RBM10* in LUAD are, at least in large part, mediated by splicing regulation, and the effects of LUAD-associated *RBM10* mutations can be predicted from their effects on the splicing of *RBM10* target genes.

## Discussion

Large-scale sequencing studies in cancer have generated data concerning genetic mutations, gene expression, and epigenetic alterations for various types of cancer. These data are valuable resources for both basic and clinical cancer investigations^6^,^38^. Predicting the consequences of functionally uncertain genetic mutations is often aided by integrative analysis, in particular by integrating information about mutations with gene expression data^10^,^39^. As described in our study, the mutational architecture and corresponding patterns of RNA expression revealed by integrative data analysis support a role of *RBM10* as a tumor suppressor in LUAD.

In our study, LUAD samples with *RBM10* PTVs showed dramatically lower *RBM10* expression levels compared to both LUAD samples lacking *RBM10* mutations or CNAs and tumor-matched normal tissues, the former of which displayed higher *RBM10* expression levels relative to the latter (Fig. 2a). These observations suggest that *RBM10* PTVs contributes to LUAD primarily via mutation induced decreases in RNA expression, which often indicates LOF. It should be noted that PTVs can also produce truncated proteins with toxic functions rather than protein LOF^40^, particularly for PTVs that do not show prominent reductions in RNA expression. For this reason, experiments are often required to determine the effects of PTVs. In addition, our observation that oncogene-negative *RBM10* coding variants reduced its mRNA expression (Fig. 3a), thereby potentially producing LOF, provided a compelling reason to investigate the underlying molecular mechanism, as shown in the case of *RBM10* c.1247A>T mutation (Fig. 3b,c).

Using our functional assessment pipeline, we successfully demonstrated distinct functional outcomes for four *RBM10* missense mutations and confirmed LOF as the functional outcome for one nonsense mutation and one frameshift mutation (Figs 4 and 5). The fact that the true functional consequences of the four missense mutations could not be predicted by the FATHMM algorithm^34^,^35^ or by measurements of RNA and protein expression demonstrates the usefulness of our pipeline.

Importantly, the observation that the effects of these missense mutations on LUAD cell proliferation (Fig. 5) correlated with their effects on splicing (Fig. 4e and Supplementary Figs S4 and S8) suggests that functional consequences of *RBM10* mutations can be predicted based on splicing of *RBM10* target genes. Of note, overexpression of one mutant (I316F) showed an enhanced inclusion of *NUMB* exon 9 and increased rate of A549 cell proliferation compared with overexpression of wild type *RBM10* (Figs 4e and 5 and Supplementary Fig. S8), suggesting a possible contribution to LUAD via change-of-function. Given the high concordance between alterations of splicing in endogenous target genes and the *NUMB* minigene reporter (Fig. 4e and Supplementary Figs S4 and S8), we propose that examining the effects of *RBM10* mutations on the splicing of *NUMB* exon9 minigene in HEK293 cells by itself may be sufficient for predicting functional consequences of the mutations. Use of our pipeline for the analysis of additional *RBM10* mutations in LUAD and other cancers may provide supporting evidence to this proposal.

EGFP fusion proteins are commonly used for evaluating the expression, subcellular localization and other functional aspects of genetic variants because they allow direct and dynamic observations. It should be noted, however, that fusion with EGFP can potentially interfere with protein function. In our study, we confirmed that *RBM10*-EGFP retained normal functions of *RBM10* by assessing subcellular localization and splicing of *RBM10* target genes (Fig. 4c and Supplementary Fig. S2). These results support the argument that this fusion protein is appropriate for investigating the properties of *RBM10* mutations.

Genetic mutations within or close to core splicing *cis*-elements (e.g. 5′ and 3′ splice sites) of cancer genes have been found to promote tumorigenesis by disrupting splicing^41^,^42^,^43^,^44^,^45^. For mutations within core *cis*-elements, it is straightforward to predict changes in splicing^10^,^39^. However, it is more difficult to predict the effects of mutations located close to core *cis*-elements^46^. Again, quantifying exon splicing patterns and gene expression levels using RNA-seq is of great help in elucidating splicing effects^10^,^39^. In addition, minigene splicing reporter assays are also useful for determining the effects of mutations on splicing, as shown by our analysis of the oncogene-negative c.1247A>T mutation of *RBM10* (Fig. 3b,c) and examples from other studies^47^,^48^.

Our results, together with those in previous investigations^10^,^18^,^27^,^28^, suggest *RBM10* and its target genes as attractive candidates for intervention in LUAD. For example, given that *RBM10* depletion can activate NOTCH signaling by promoting the formation of the long isoform of *NUMB*, it is possible that NOTCH inhibitors or specific modulators of *NUMB* splicing may be helpful for LUAD patients with *RBM10* LOF mutations. Because *RBM10* regulates the splicing of hundreds of genes^26^, further studies are required to fully elucidate its functional and regulatory roles in LUAD. These analyses can be expected to provide additional opportunities for clinical translation.

In conclusion, our study revealed that *RBM10* mutations contribute to LUAD via genetic mutation and subsequent splicing deregulation. The methods we employed should be useful for the functional characterization of additional *RBM10* mutations and mutations in other cancer-associated splicing regulators.

## Methods

### LUAD data in Public databases

LUAD mutation and copy number alteration (CNA) data were downloaded from the Catalogue of Somatic Mutation in Cancer (COSMIC) website http://cancer.sanger.ac.uk/cosmic)^30^. LUAD mutation and expression data were obtained from The Cancer Genome Atlas (TCGA) website (http://cancergenome.nih.gov/). Data concerning *RBM10* mutations, CNA and its RNA expression levels from these databases were extracted and combined. RNA expression levels in TCGA were estimated from RNA-Seq data using the “RNA-Seq by Expectation Maximization” (RSEM) algorithm and presented as “normalized counts” following upper quartile normalization^49^,^50^. *RBM10* mRNA expression levels in each group of LUAD samples with different types of *RBM10* mutations or CNAs were compared to expression levels in samples lacking *RBM10* mutations or CNAs or normal tissues adjacent to tumors, respectively. Survival analysis of LUAD patients was performed using data deposited in the Kaplan Meier plotter online database (http://www.kmplot.com)^31^. All the parameters were set as “default” in the online tool for lung cancer analysis, except for the following specifications. We used Affy ID: “215089_s_at” as the JetSet probe set (recommended by the online tool as the best probe set), selected “Auto select best cutoff”, and chose Histology: “lung adenocarcinoma.”

### Cell culture

Human embryonic kidney (HEK) 293 and HEK293-FT cells were cultured in Dulbecco’s Modified Eagle’s medium (DMEM) supplemented with 10% fetal bovine serum (FBS). The human lung adenocarcinoma cell line A549 was cultured in F-12K medium supplemented with 10% FBS. All cultures were maintained under standard culture conditions (37 °C, 5% CO_2_).

### Constructs

*RBM10*-EGFP was generated by amplifying *RBM10* from pFRT-TO-*RBM10*^26^, followed by double digestion of the PCR Product with *Nhe*I and *Eco*RI (NEB) and ligation into *Nhe*I/*Eco*RI linearized pEGFP-N3 vector (Clontech) using T4 DNA ligase (NEB). The tet-on lentiviral plasmids carrying *RBM10*-EGFP were constructed by amplifying the target sequence from *RBM10*-EGFP, cutting the resulting PCR product with *Not*I/*Mlu*I and ligating this to *Not*I/*Mlu*I linearized pLVX-Tight-Puro Vector (Clontech).

Two minigene splicing reporters were constructed. For the *RBM10*-E12M minigene vector, genomic segments from the *RBM10* exon 11 to exon 13 (chrX: 47039611–47040800, GRCh37) were amplified from HEK293 genomic DNA and inserted into the pcDNA3.1(-) expression vector (Invitrogen) using the Clone Express II reagent (Vazyme) following the manufacturer’s protocol. For the *NUMB*-exon9 minigene, DNA fragments containing *NUMB* exon 8, intron 8, exon 9, intron 9 and the first 100 bp of exon 10 (chr14: 73743902–73749213, GRCh37) were amplified and inserted into pcDNA3.1(-) using the Gibson Assembly Cloning Kit (NEB) following instructions in the manufacturer’s manual.

### Mutagenesis

Mutations were introduced into the *RBM10*-EGFP plasmid and *RBM10*-E12M minigene using Q5 Site-Directed Mutagenesis Kit (NEB) or Mut Express II Fast Mutagenesis Kit V2 (Vazyme) following the manufacturer’s instructions.

### Plasmids and siRNAs transfection

For plasmids, cells were seeded in 6-well plates at approximately 50% confluency and transfected with 1 μg total plasmid DNA per well using Lipofectamine 3000 reagent (Thermo Scientific). For siRNAs, cells were transfected with Lipofectamine RNAiMax reagent (Thermo Scientific) using the reverse transfection method according to the manufacturer’s instructions. Cells were harvested 24 hours for minigene splicing reporters and 48 hours for *RBM10* expression plasmids after transfection. The harvested cells were used for RNA and/or protein extraction.

### Total RNA preparation, reverse transcription and PCR (RT-PCR)

Total RNA was extracted from cell lysates in TRIzol (Invitrogen) and treated with DNase I using the TURBO DNA-free kit (Ambion). 1 μg DNase I-treated total RNA from each sample was reverse transcribed using HiScript II Reverse Transcriptase (Vazyme) and oligo dT_20_ primers. *RBM10* mRNA expression level and splicing changes of target genes were assessed by semi-quantitative RT-PCR as described previously^26^. The RT products from minigene splicing reporters were amplified by gene-specific forward primer and vector specific reverse primer. *GAPDH* was used as an internal control. All PCR primers were listed in Supplementary Table S2. PCR products were mixed with loading buffer containing GelRed fluorescent DNA stains (GENEray), resolved in 1.5 or 2% agarose gels and visualized using a UV image system. The signal intensity of each band was quantified using Image J software (https://imagej.nih.gov/ij/). The percent-spliced-in (PSI) value was defined as: PSI = (intensity of the upper band)/[intensity of the upper band + (L_L_/L_S_) × (intensity of the lower band) × 100%, where L_L_ and L_S_ represent the lengths of the long and short PCR products that include or exclude the alternative exon, respectively.

### Western blotting

Western blot analysis was performed as previously described^26^. Briefly, cells were lysed in RIPA buffer (25 mM Tris-HCl, pH 7.4, 150 mM NaCl, 1% NP-40, 0.1% SDS and 0.1% sodium deoxycholate) followed by brief sonication. Lysates were cleared by centrifugation and the supernatant fractions with added loading buffer were boiled at 95 °C for 5 min. Denatured protein lysates were resolved by SDS-PAGE and transferred to a PVDF membrane (Millipore). The membranes were blocked with 5% milk in TBST (50 mM Tris-HCl, pH 7.4, 150 mM NaCl, 0.1% Tween 20) and sequentially probed with primary antibodies and horseradish peroxidase (HRP)-conjugated secondary antibodies. Bands were detected with an enhanced chemiluminescence (ECL) system (Millipore) and analyzed using the ChemiDoc^TM^ XRS+ image system (BioRad). The following antibodies were used: rabbit anti-*RBM10* (cat. No. HPA034972, 1:10000, Sigma), rabbit anti-α-tubulin (cat. No. 11224-1-AP, 1:5000, proteintech), Goat anti-rabbit IgG-HRP (cat. No. 458, 1:5000, MBL).

### Fluorescence imaging

Cells grown on coverslips were fixed in 4% PFA and stained with 1 μg/ml DAPI (Invitrogen). Fluorescent images were acquired using a Leica DM IL LED fluorescent microscope with a 40x objective and DFC450 C camera (Leica) and processed using Image J software (https://imagej.nih.gov/ij/).

### Generation of tet-on *RBM10*-EGFP A549 cell lines

Inducible overexpression of *RBM10*-EGFP wild type (WT) or mutants (MUTs) in A549 cells was achieved using the Lenti-X Tet-On Advanced Inducible Expression System (Clontech) according to the manufacturer’s manual. Briefly, lentiviral plasmids (pLVX-tight-puro) carrying *RBM10*-EGFP WT or MUTs or TetR (pLVX-Tet-On) were transfected into HEK293-FT cells together with three packaging plasmids (pLP1, pLP2, and pLP/VSVG, Invitrogen). Medium containing the lentiviral particles was harvested 48 or 72 hrs after transfection.

Lentiviral particles carrying TetR was added into the A549 cell cultures and selected by 200 μg/ml G418 (Invitrogen). The G418 selected cells were transfected with lentiviruses carrying *RBM10* WT or MUTs followed by selection in medium containing 2 μg/ml puromycin (Sigma). Stably transfected A549 cells were maintained under standard conditions and overexpression of *RBM10*-EGFP proteins were induced by 1 μg/ml doxycycline treatment (Sigma) for 2 days unless otherwise stated.

### Cell proliferation assays

Tet-on *RBM10*-EGFP A549 cells were seeded in 96-well plates (1000 or 1500 cells/well) and treated with 1 μg/ml doxcycline to induce the overexpression of WT or MUTs. Cells not exposed to doxcycline were used as controls. Numbers of viable cell were measured each day for 5 days using Cell Counting Kit-8 (CCK-8) following the manufacturer’s manual (Dojindo). For each measurement, cells were incubated with CCK-8 reagents for 1 hour at 37 °C and the optical absorption at 450 nm was measured using a spectrophotometer (MULTISKAN MK3, Thermo Scientific). For each time point and condition, we included 4 wells of cells seeded from the same set of cultured cells as technical replicates. CCK-8 assays were carried out three times using three independently prepared sets of cells as biological replicates. The number of viable cells is proportional to the absorption values.

### Statistical analysis

Statistical analyses were carried out using tests when the data satisfied the assumptions of the tests. For comparisons of *RBM10* mRNA levels in LUAD samples, *P* values were calculated by Mann-Whitney tests followed by Benjamini & Hochberg corrections for multiple comparisons using the open source statistical programming environment ‘R’. For experimental data, statistical tests were carried out using GraphPad Prism 6 (GraphPad software). For comparisons of sample pairs, two-tailed Student’s *t* tests were used. For experiments with one variable and multiple conditions, one-way ANOVA followed by Dunnett’s multiple comparisons test was used. For experiments with two independent variables, two-way ANOVA followed by Bonferroni correction for multiple testing was used.

### Primer and oligonucleotide

All primer and oligonucleotide sequences are listed in Supplementary Table S2.

## Additional Information

**How to cite this article**: Zhao, J. *et al*. Functional analysis reveals that *RBM10* mutations contribute to lung adenocarcinoma pathogenesis by deregulating splicing. *Sci. Rep.* **7**, 40488; doi: 10.1038/srep40488 (2017).

**Publisher's note:** Springer Nature remains neutral with regard to jurisdictional claims in published maps and institutional affiliations.

## Supplementary Material

Supplementary Figures and Table S2

Supplementary Table S1
