# Supplementary material for: Functional analysis reveals that RBM10 mutations contribute to lung adenocarcinoma pathogenesis by deregulating splicing
Source: Sci Rep. 2017 Jan 16;7:40488. doi: 10.1038/srep40488 (PMC5238425; doi:10.1038/srep40488)
Supplement: Supplementary Figures and Table S2 [file srep40488-s1.pdf]

Supplementary Information

Functional analysis reveals that RBM10 mutations contribute to lung adenocarcinoma pathogenesis by deregulating splicing

Jiawei Zhao[1,+], Yue Sun[2,3,+], Yin Huang[6], Fan Song[1], Zengshu Huang[5], Yufang Bao[1], Ji Zuo[1], David Saffen[1,3,4], Zhen Shao[6], Wen Liu[1,*], Yongbo Wang[1,*]

[1] Department of Cellular and Genetic Medicine, School of Basic Medical Sciences, Fudan University, Shanghai, 200032, China

[2] School of Life Sciences, Fudan University, Shanghai, 200438, China

[3] Institutes of Brain Science, Fudan University, Shanghai, 200032, China

[4] State Key Laboratory for Medical Neurobiology, Fudan University, Shanghai, 200032, China

[5] Shanghai Medical College, Fudan University, Shanghai, 200032, China

[6] Key Laboratory of Computational Biology, CAS-MPG Partner Institute for Computational Biology, Shanghai Institutes for Biological Sciences, Chinese Academy of Sciences, Shanghai 200031, China

[+] These authors contributed equally to this work.

* Corresponding authors.

Corresponding authors

Correspondence to Yongbo Wang, E-mail: wangyongbo@fudan.edu.cn or Wen Liu, E-mail: liuwen@shmu.edu.cn

Supplementary Figures

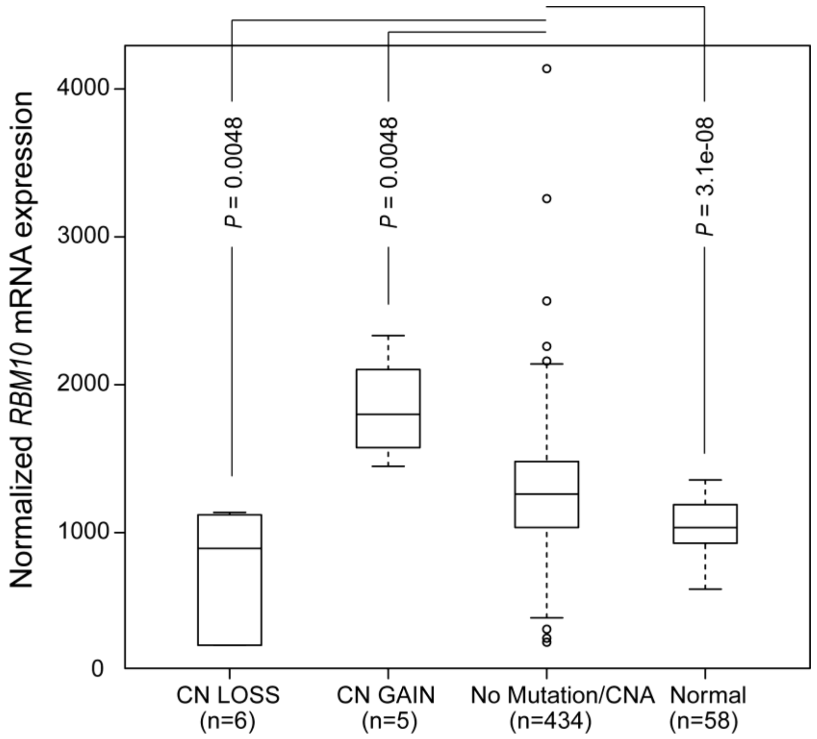

Supplementary Figure S1. Effects of RBM10 copy number alterations (CNAs) on its RNA expression. Box plot shows *RBM10* mRNA expression in LUAD samples with RBM10 copy number loss (CN LOSS), copy number gain (CN GAIN), without RBM10 mutation and CNA (No Mutation/CNA), and in tumor-adjacent normal tissue (Normal). *P* values were calculated using Mann-Whitney tests followed by Benjamini & Hochberg corrections. Data are from TCGA and COSMIC (see Methods for details). Boxes represent the medians (inside lines) and the quartiles (upper and bottom boarder lines). "Whiskers" above and below the boxes represent maximum and minimum values within the 1.5 × IQ (inter quarter) range.

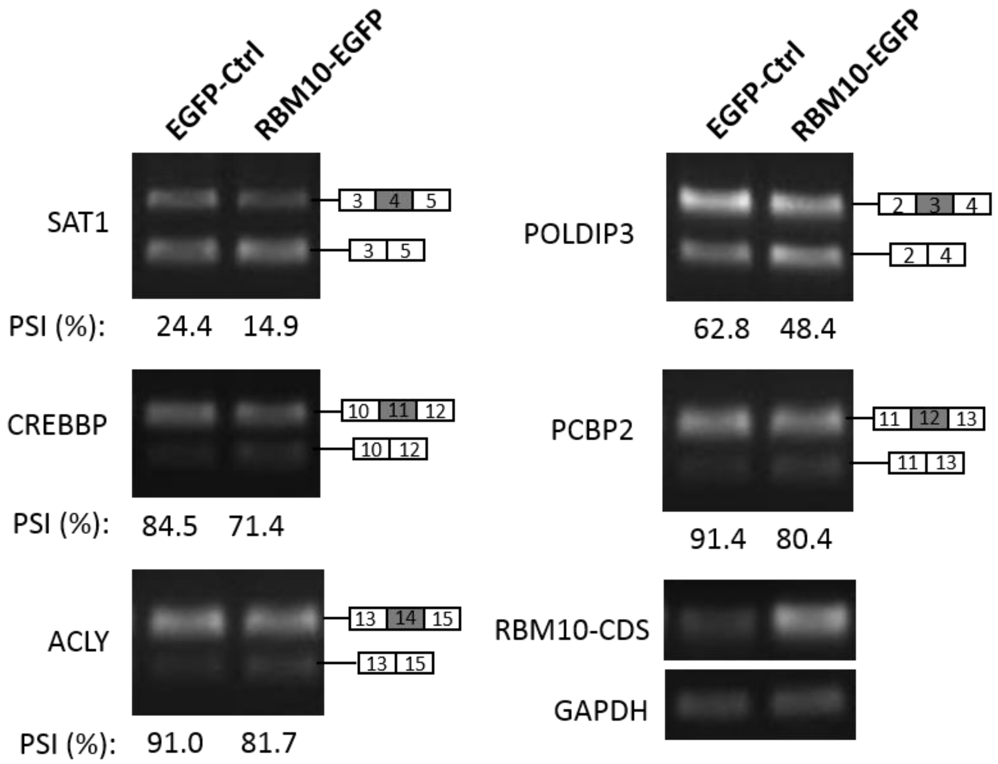

Supplementary Figure S2. RBM10-EGFP overexpression (OE) promotes exon skipping in RBM10 target genes in HEK293 cells. Differences in splicing in five RBM10 target genes transiently overexpressing EGFP or RBM10-EGFP were detected by RT-PCR. Shown are gel images of RT-PCR products, whose identities are indicated on the right, based on Refseq transcript variant 1 sequences of these genes. RT-PCR using primers binding to RBM10 coding sequences (RBM10-CDS) provided a measure of the extent of OE. *GAPDH* was used as an internal control. Exon inclusion levels, represented by PSI (percent-splice-in) values, were calculated from the PCR band intensities quantified using ImageJ software.

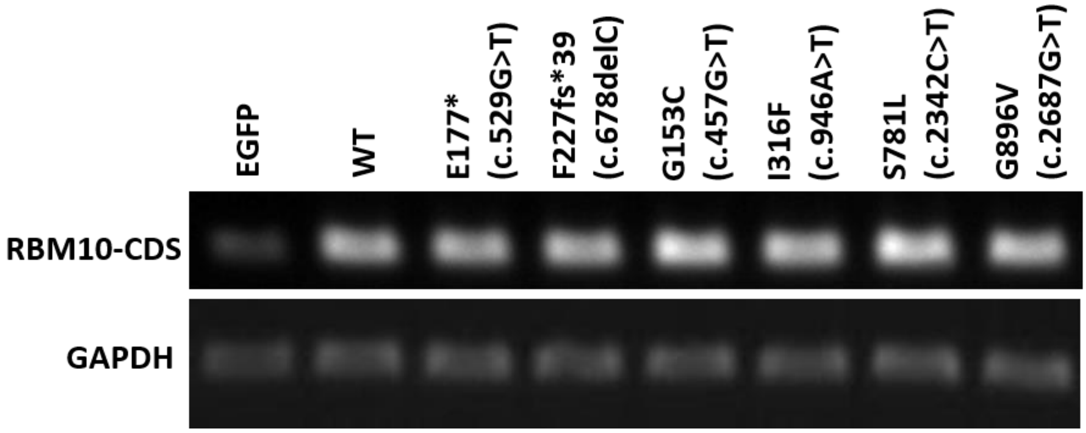

Supplementary Figure S3. RT-PCR assessment of *RBM10* mRNA expression levels in RBM10-EGFP wild type (WT) or mutant (MUT) transfected HEK293 cells. RT-PCR primer binding to RBM10 coding sequence (RBM10-CDS) was used to measure the extent of OE. A representative gel image from three independent experiments is shown. *GAPDH*: internal control.

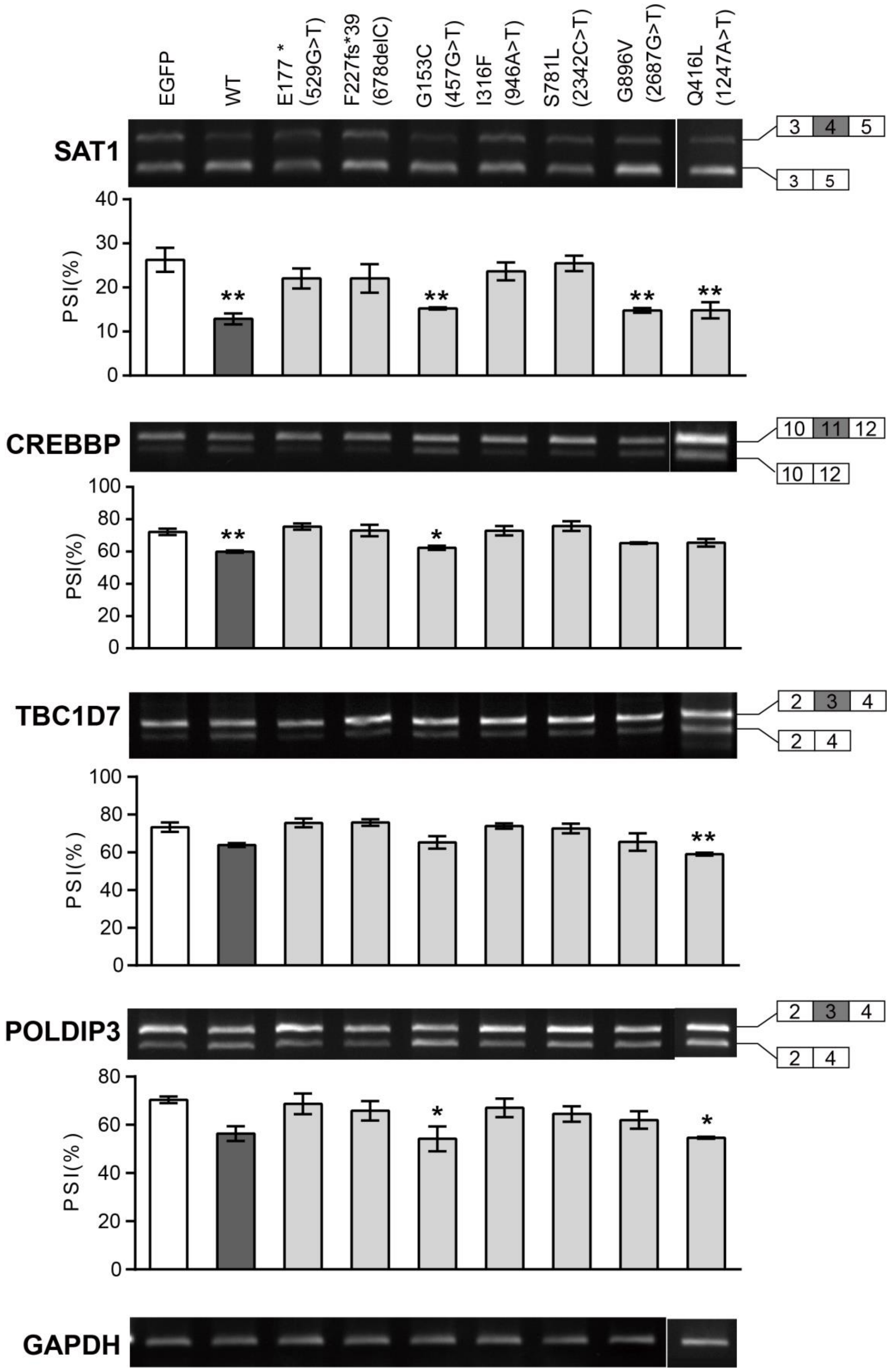
EGFP
WT
E177 * (529G>T)
F227fs*39 (678delC)
G153C (457G>T)
I316F (946A>T)
S781L (2342C>T)
G896V (2687G>T)
Q416L (1247A>T)
SAT1
3 4 5
3 5
PSI(%)
**
**
**
**
CREBBP
10 11 12
10 12
PSI(%)
**
*
TBC1D7
2 3 4
2 4
PSI(%)
**
POLDIP3
2 3 4
2 4
PSI(%)
*
*
GAPDH

Supplementary Figure S4. Splicing changes in four RBM10 target genes induced by RBM10-EGFP wild type (WT) and mutants (MUTs) overexpression in HEK293 cells. Splice variants were examined by RT-PCR and quantified as PSI (percent-splice-in) values. Means ± SEM of PSIs from three independent experiments are plotted below representative gel images. *GAPDH*: internal control. All samples were compared with EGFP controls. ns: not significant, * $P \leq 0.05$, ** $P \leq 0.01$ (One-way ANOVA followed by Dunnett's test for multiple comparisons to control). Note that RT-PCR products for Q416L were prepared in parallel with the other samples, but resolved in non-adjacent lanes in the gels.

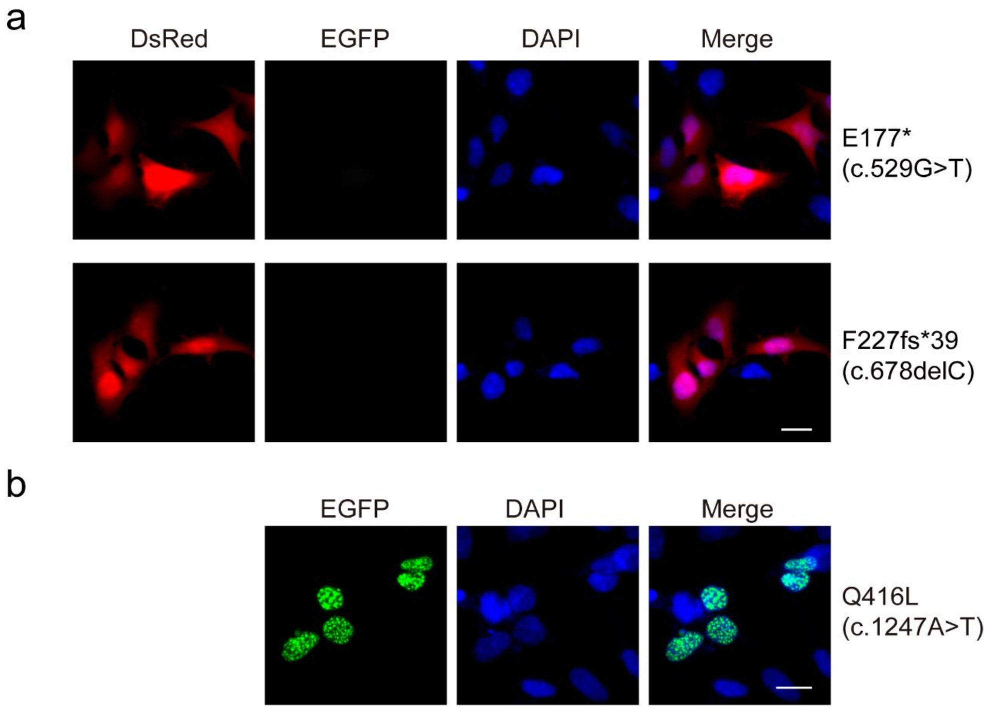

Supplementary Figure S5. Subcellular localizations of RBM10-EGFP E177*, F227fs*39 and Q416L in HEK293 cells. (a) RBM10-EGFP E177* and RBM10-EGFP F227fs*39 were cotransfected with DsRed expression plasmids. RBM10-EGFP fluorescence was absent in these cells, consistent with lack of expression of the fusions proteins. **(b)** RBM10-EGFP Q416L localized to the nucleus. Magnification: 40x. Scale bar: 10 μm. Nuclei were visualized by DAPI staining.

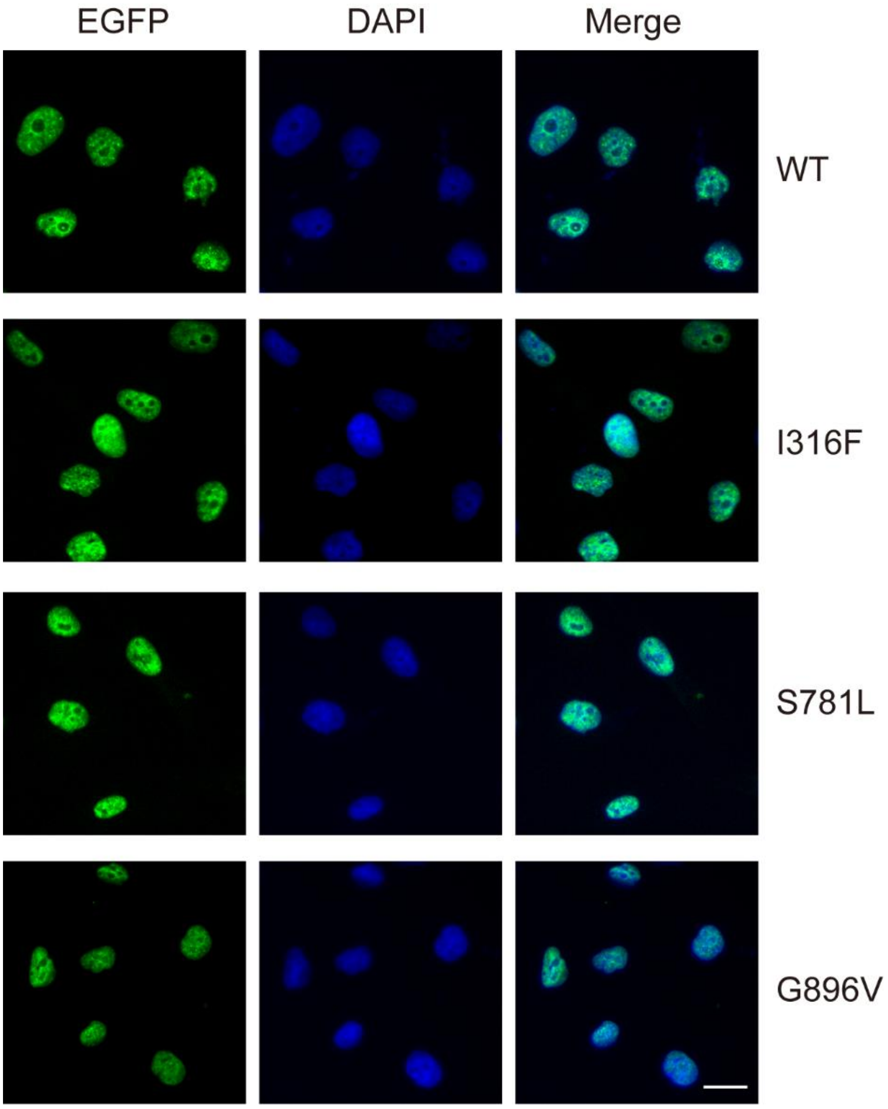

Supplementary Figure S6. Nuclei subcellular localizations of RBM10-EGFP wild type (WT), I316F, S781L and G896V fusion proteins in tet-on A549 cells. Magnification: 40x. Scale bar: 10 μm. Nuclei were visualized by DAPI staining.

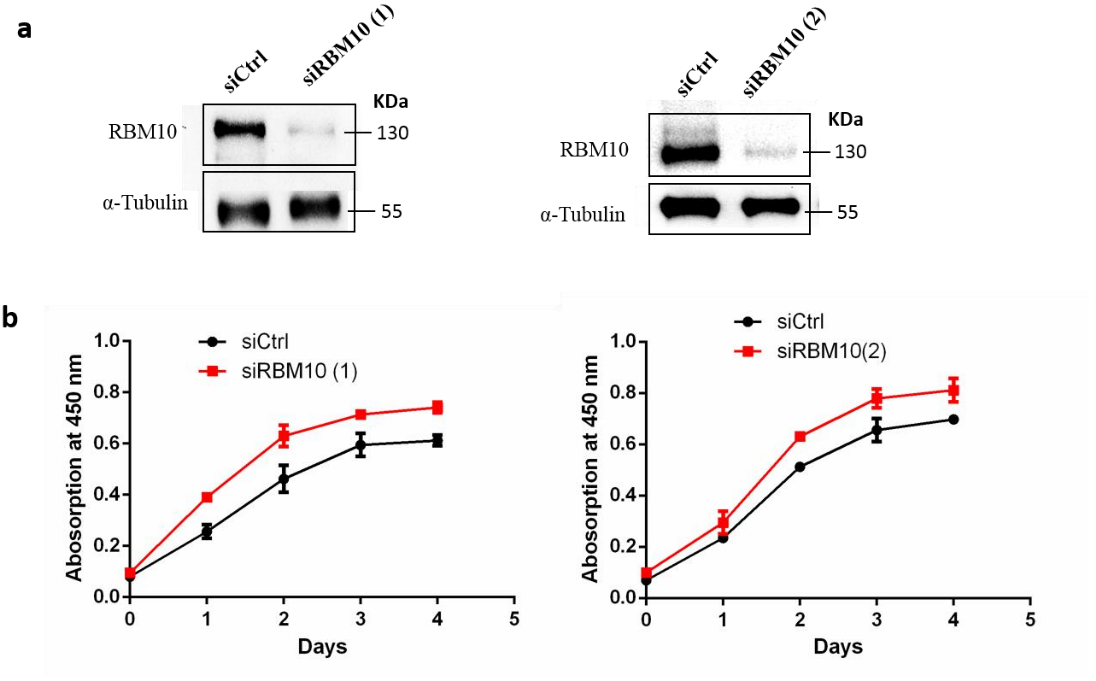

Supplementary Figure S7. RBM10 depletion promotes proliferation of LUAD A549 cells. (a) Western blots showing the degree of RBM10 depletion achieved by transfecting A549 cells with siRNAs against RBM10 [siRBM10(1) or siRBM10(2)] or non-target control siRNA (siCtrl). Sequences of siRBM10(1), siRBM10(2) and control siRNA are listed in Supplementary Table S2. α-Tubulin served as the loading control. **(b)** CCK8 analysis of the proliferation rates of A549 cells transfected with siRBM10(1), siRBM10(2) or siCtrl. Error bars: ± SD, n = 4 technical replicates.

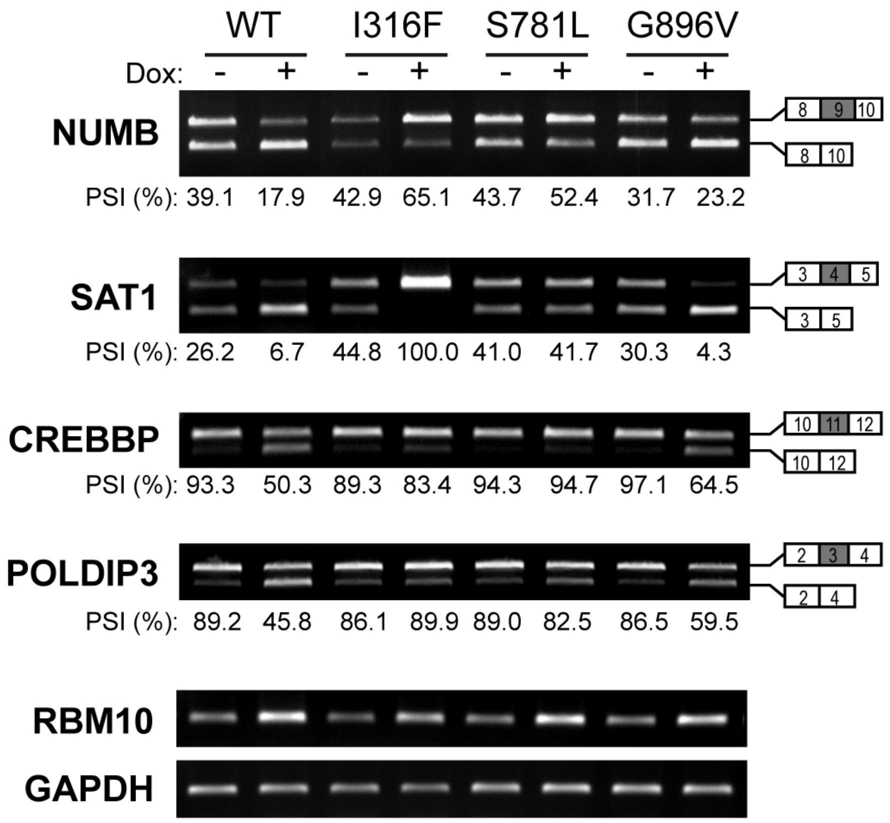

Supplementary Figure S8. Splicing changes of four RBM10 target genes induced by overexpression (OE) of RBM10 wild type (WT) or one of three missense mutants (MUTs) in tet-on A549 cells. OE was achieved by 1 μg/ml doxycycline (Dox) treatment. Splice variants were examined by RT-PCR and quantified as PSI (percent-splice-in, %) values and shown below the gel image. Primer binding to RBM10 coding sequence (RBM10-CDS) measures the extent of OE. *GAPDH*: internal control.

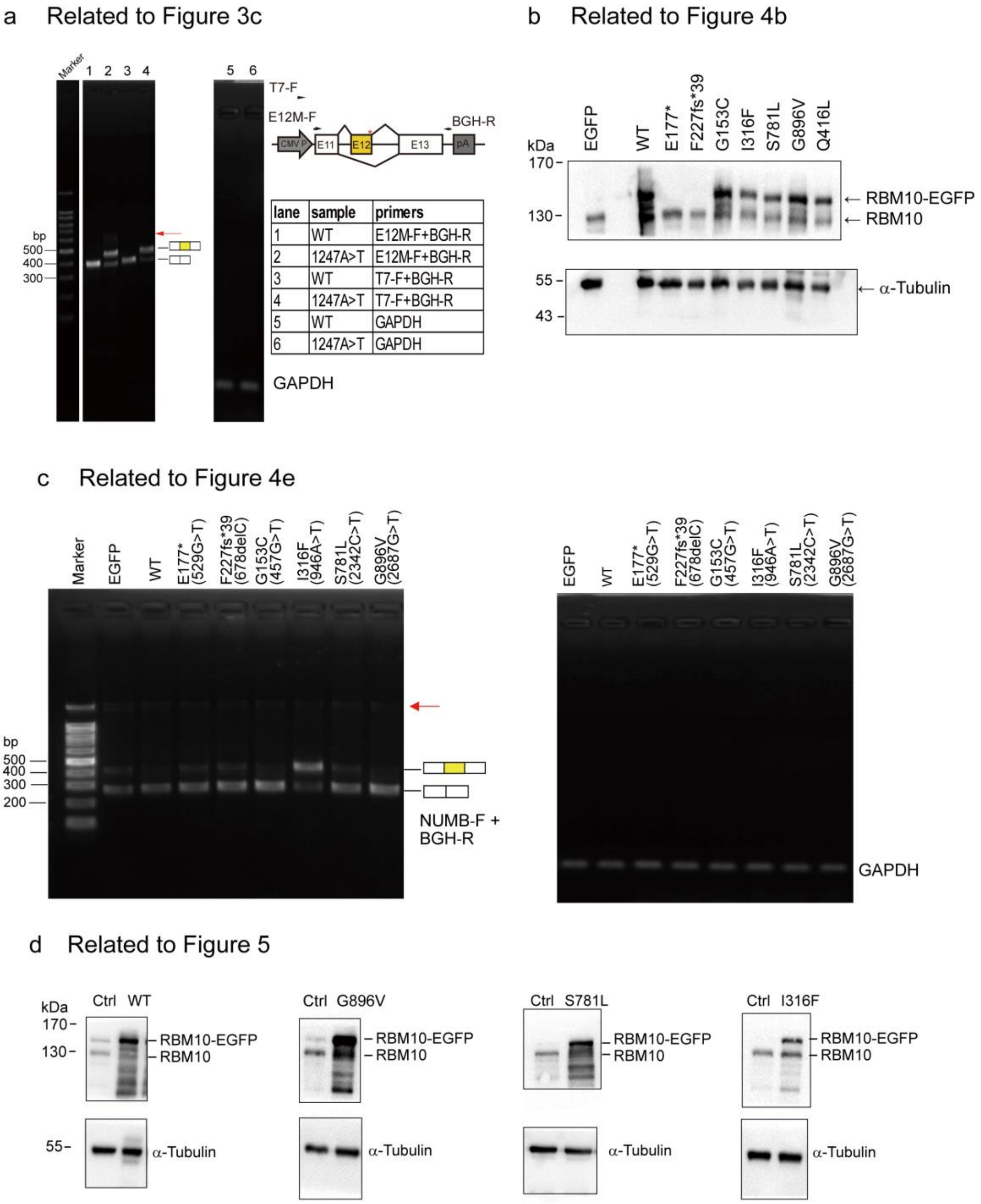

Supplementary Figure S9. (a-d) Full-length images of agarose gels and western blots related to Figure 3c, 4b, 4e and 5, respectively. PCR products by primer T7-F and BGH-R were included in (a) to support the specificity of those by primer E12M-F and BGH-R. The band marked by red arrow in (a) is likely representing *RBM10* intron 11 unspliced product, and that

in (c) is likely representing *NUMB* intron 9 unspliced product. The nonspecific bands detected in (d) are likely degraded products or artifacts of RBM10-EGFP overexpression. bp: base pair. Marker: 100 bp DNA ladder from NEB.

Supplementary Tables

Supplementary Table S1. RBM10 mutations and mRNA expression levels in COSMIC and TCGA LUAD samples. Normalized *RBM10* mRNA expression levels were obtained from TCGA. FATHMM prediction results were obtained from COSMIC. Four reported oncogene-negative samples were highlighted in yellow. Table S1 is provided as a separate excel file.

Supplementary Table S2. Sequences of oligonucleotides and PCR primers used in this study.

**Construct**		
**Primer Name**	**Primer Sequence (5'-3')**	**Vector**
R10_ NheI_F	ACGCGCTAGCATGGAGTATGAAAGACGTGGTG	RBM10-EGFP
R10_ EcoRI _R	ATTTGAATTCCTCTGGGCCTCGTTGAAGCG	
R10-NotI-F	TAATGCGGCCGCGGTCTATATAAGCAGAGCTGGT	pLVX-RBM10-GFP
GFP-MluI-R	GTCACGCGTTGATTATGATCTAGAGTCGCGG	
RBM10-E12M-F	GGGAGACCCAAGCTGGCTAGCGAGGCAGCCCAGCTGCTGC	RBM10-E12M minigene
RBM10-E12M-R	TAGTCCAGTGTGGTGGAATTCCTGCTCCAGTGGGATCCCCTTTG	
NUMB-1_fwd	tttaaacgggccctctagacTGCCAGAAGTAGAAGGGG	NUMB minigene
NUMB-1_rev	aatgtgtaagTGCTCAATAAATGGTGCC	
NUMB-2_fwd	ttattgagcaCTTACACATTGCTTGCCAC	
NUMB-2_rev	gagtcagtgcCATTAGCTACAACGGGAG	
NUMB-3_fwd	gtagctaatgGCACTGACTCAGCCTTCC	
NUMB-3_rev	tgatcagcggtttaaacttaACCTCTTCTAACCATCGGTC	

**Mutagenesis**	
**Primer Name**	**Sequence (5'-3')**
E12M- A1247T-F	CCATCTCACTGgtactcagACCCCTTGTGCCTCCCAGC
E12M- A1247T-R	ctgagtacCAGTGAGATGGCCCACTGGGCCGCAGCAA
457G-T (G153C)-F	AGTCGCACtGCGTGCAAGCAC
457G-T (G153C)-R	GCAGCTGGCCACGGATGTCATCC
529G-T (E177)-F	CCTTCGTCtAGTTTAGTCACTT
529G-T (E177-)-R	CGAAGCCCCGGCTCTGA
RBM10-C678del-F	CGTCCAGAATTCAAACGCCGAGAGAAGTGCTTCAAATG
RBM10-C678del-R	GCGTTTGAATTCTGGACGCCACACTTATTGCACAGC

946A-T (I316F)-F	TGGATTCCtTCCTGGGGGCC	
946A-T (I316F)-R	TGGTGCTGTGTGGGTTCAGGT	
RBM10-A1247T-F	CCATCTCACTGGCCTCCCAAGGTGGGGAGGGTACCTG	
RBM10-A1247T-R	TGGGAGGCCAGTGAGATGGCCCACTGGGCCGCAGC	
2342C-T (S781L)-F	CAGCTCTtAGGGCTCCAC	
2342C-T (S781L)-R	CTGGTGCCGGATGAGCG	
2687G-T (G896V)-F	TGCGGGtCTCCGGCCTGG	
2687G-T (G896V)-R	CCCGTGTTTGGGCCTCGATAGGCG	
**RT-PCR**		
**Primer Name**	**Sequence (5'-3')**	**Product length (bp)**
ACLY_F	CTGCAAAGAAGGCCAAGCC	141, 171
ACLY_R	CGTCTCGGGAGCAGACATAG	
CHTOP_F	GCCCAGCAGATGGAGAATAG	534, 212
CHTOP_R	AGCATCCAGGTGTCCTTTTG	
CREBBP_F	AGGCACAACCTGTGAGACCT	142, 187
CREBBP_R	ACTGAGCCCATGCTGTTCAT	
NUMB_F	GAAGTAGAAGGGGAGGCAGA	225, 369
NUMB_R	GTCGGCCTCAGAGGGAGTA	
PCPB2_F	TTGACCAAGCTGCACCAG	129, 168
PCBP2_R	ATCGTTTGGAATGGTGAGTTC	
POLDIP3-F	TCGAATCAAAGGGAAAGTGC	273, 360
POLDIP3-R	CCTGAGGCTGCAAACTTCAT	
SAT1_F	TTTGGAGAGCACCCCTTTTA	202, 92
SAT1_R	ATGGCAAAACCAACAATGCT	
TBC1D7_F	TGAGAAAGTGGGGTTTCGTG	337, 256
TBC1D7_R	TGGAAAAGAGGGACTTCGAG	
RBM10-CDS-F	GCCTCTACTATGACCCCAACTCCCA	82
RBM10-CDS-R	GTCCGCCTCTCCCCATCCCA	
GAPDH-F	TGCACCACCAACTGCTTAGC	87
GAPDH-R	GGCATGGACTGTGGTCATGAG	
BGH-R	TAGAAGGCACAGTCGAGG	/
**siRNAs**		
**Oligo Name**	**Sequence (5'-3')**	
siCtrl	r(UUCUCCGAACGUGUCACGU)dTdT	
siRBM10-1	r(CCGCUGUGCUCAAAUCUGA)dTdT	
siRBM10-2	r(CUUCGCCUUCGUCGAGUUUAG)	
